# Supplementary material for: EnzML: multi-label prediction of enzyme classes using InterPro signatures
Source: BMC Bioinformatics. 2012 Apr 25;13:61. doi: 10.1186/1471-2105-13-61 (PMC3483700; doi:10.1186/1471-2105-13-61)
Supplement: Addtional file 5 — The Java code to format the data files, evaluate and predict. The file enzml_java_code.tar.gz contains the Java code used to format database data to ARFF and XML formats, to execute cross and train-test (jackknife) evaluations and to record evaluation results to database. More information is included in the readme.txt file and the Javadoc files. The code can be used with a MySQL database. To use a different database software, other JDBC drivers might be required. [file 1471-2105-13-61-S5.gz › java_code/enzml2011/doc/uk/ac/ed/inf/taweka/mulan/learn/class-use/TrainTestExperimenterSerialized.html]

Uses of Class uk.ac.ed.inf.taweka.mulan.learn.TrainTestExperimenterSerialized


---


|  |  |  |
| --- | --- | --- |
| **Overview** \| **Package** \| **Class** \| **Use** \| **Tree** \| **Deprecated** \| **Index** \| **Help** | |  |
| PREV   NEXT | **FRAMES**    **NO FRAMES**     **All Classes** |  |


---


## **Uses of Class uk.ac.ed.inf.taweka.mulan.learn.TrainTestExperimenterSerialized**

| Packages that use TrainTestExperimenterSerialized | |
| --- | --- |
| **uk.ac.ed.inf.taweka.test.mulan.learn** |  |

Uses of TrainTestExperimenterSerialized in uk.ac.ed.inf.taweka.test.mulan.learn

| Methods in uk.ac.ed.inf.taweka.test.mulan.learn that return TrainTestExperimenterSerialized | |
| --- | --- |
| `static TrainTestExperimenterSerialized` | `TrainTestExpSerializedTest.getTrainTestExpSerialized100()` |
| `static TrainTestExperimenterSerialized` | `TrainTestExpSerializedTest.getTrainTestExpWithSerializedModel(int id, java.lang.String trainedModelPath)` |

---


|  |  |  |
| --- | --- | --- |
| **Overview** \| **Package** \| **Class** \| **Use** \| **Tree** \| **Deprecated** \| **Index** \| **Help** | |  |
| PREV   NEXT | **FRAMES**    **NO FRAMES**     **All Classes** |  |


---
